# SMYD proteins in immunity: dawning of a new era

**DOI:** 10.3934/biophy.2016.4.450

**Published:** 2016-10-17

**Authors:** Maysaa Doughan, Nicholas Spellmon, Chunying Li, Zhe Yang

**Affiliations:** 1 Department of Biochemistry and Molecular Biology, Wayne State University School of Medicine, Detroit, MI, USA; 2 Center for Molecular and Translational Medicine, Georgia State University, Atlanta, GA, USA

In the past decade, the SMYD protein family has gradually become a center of research, thanking for its essential role in heart and muscle development and cancer development. However, because of such a role, the research scope for the SMYD protein family has been fairly stereotyped, its focus largely restricted to muscle and cancer. Initial studies of SMYD1 and SMYD3 were responsible for these biased research directions. SMYD1 was originally identified as a myogenic factor and regulated cardiomyocyte maturation and proper formation of the right ventricle [1]. SMYD3 was initially identified as an oncogene and regulated expression of cell-cycle genes in colorectal and hepatocellular carcinomas [2]. These findings have clearly indicated an essential role of SMYD1 and SMYD3 in cardiac muscle development and tumorigenesis respectively. Because of this, the research directions in SMYD proteins have been strongly influenced by these two studies, and much subsequent research has biased towards muscle and cancer.

The function of SMYD2, SMYD3, and SMYD4 was studied in muscle. SMYD2 was found to regulate sacromeric I-band stability in cardiac muscle [3]. Knockdown of SMYD2 in zebrafish led to impaired cardiac performance and defective myofibril organization. SMYD3 was found to regulate muscle development and involved in skeletal muscle atrophy [4]. Knockdown of SMYD3 in zebrafish resulted in abnormal heart development and maturation of myogenic cells [5]. SMYD4 has also been shown to be important for muscle development. Knockdown of SMYD4 in *Drosophila* resulted in eclosion failure likely due to defective abdominal muscle development [6]. It is therefore not surprising that SMYD proteins were stereotyped as heart and muscle proteins [7].

The SMYD cancer research flourished shortly after the initial SMYD3 cancer studies. To date, SMYD3 has been found to be overexpressed in over 15 types of cancers [7]. Overexpression of SMYD3 has been repeatedly shown to promote cancer cell growth. Knockdown of SMYD3 resulted in a significant reduction in cancer cell growth. Poor cancer prognosis is often linked with SMYD3 overexpression. SMYD2 and SMYD4 have also been linked to cancer. SMYD2 is overexpressed in leukemia and esophageal squamous cell carcinoma [8,9]. Low survival rates in leukemia patients are associated with the high expression level of SMYD2. Knockdown of SMYD2 in esophageal squamous cell carcinoma inhibited tumor cell growth; with overexpression of SMYD2, promoted proliferation. SMYD4 was identified as a tumor suppressor in breast cancer [10]. Therefore, it is also not surprising that SMYD proteins have been additionally stereotyped as cancer proteins [7].

However, new discoveries are shaking the traditional view of SMYD stereotypes. Accumulating evidence is pointing out that the SMYD protein family plays an important role in immunity [11–13]. Hilariously, the first clue for such a role came from a report two decades ago that SMYD1 was highly expressed in thymus and restricted to cytotoxic T cells [14]. SMYD1 may regulate T-cell binding to antigen infected cells, as it shares a bidirectional promoter with the T-cell surface glycoprotein CD8b. SMYD1 is not expressed in antigen-independent T cell lines, suggesting a role in antigen-dependent cell growth and resting. SMYD1 contains closely-spaced interferon gamma (IFNγ) regulatory sequences and may be IFNγ -inducible. IFNγ plays many roles in regulating inflammation and T-cells activation. This indicates that SMYD1 may be involved in immunity through the IFNγ signaling pathway and serve as a downstream effector of IFNγ. However, the immunological role of SMYD proteins was not seriously considered until only recently when the first study of SMYD5 was reported. In 2012, SMYD5 was found to regulate expression of toll-like receptor 4 (TLR4) target genes during the immune response [11]. SMYD5 is significantly upregulated in macrophages upon lipopolysaccharide (LPS) challenge. Latest studies showed that SMYD2 and SMYD3 are also involved in immunity. SMYD2 was found to negatively regulate macrophage activation and M1 polarization [12]. SMYD2 expression in macrophages suppresses differentiation of T-helper 17 cells (Th17) but promotes regulatory T-cells differentiation. This suggests that SMYD2 may be involved in negative regulation of adaptive immune response. SMYD3 was found to promote formation of inducible regulatory T cells and may be involved in reducing autoimmunity [13]. Respiratory virus infection in SMYD3-deficient mice results in exaggerated lung inflammation. Current data, though still limited in scope, have clearly pointed out an essential role of SMYD proteins in immunity and inflammatory control.

Immunity and inflammation will likely become new stereotypes for SMYD proteins. New roles and new mechanisms are waiting to be explored. However, new stereotypes are not conflicting with the traditional stereotypes; instead, they are complementary and deepening SMYD research. The immunological field has been greatly facilitated by cancer studies. The mechanism of SMYD immune regulation was entirely based on the results of the cancer research. SMYD proteins were initially identified as histone lysine methyltransferases and regulated gene expression through histone methylation [7]. In cancer, SMYD3 methylates histone H3 K4 and upregulates a number of oncogenes such as c-Myc, STAT3, and MMP9 [2]. Oncogenesis is directly correlated with SMYD3 methyltransferase activity. These earlier studies have indicated epigenetic mechanisms being responsible for the SMYD3 functions. This view has been instrumental for the immunological research and helped to narrow down the mechanistic possibilities. It was proposed that SMYD proteins may be involved in immunity also through epigenetic mechanisms and directly regulating inflammatory gene expression [11]. Indeed, SMYD2 methylates H3 K36 and represses IL-6 and TNFα expression [12]. SMYD3 methylates H3 K4 and promotes Foxp3 expression [13]. SMYD5 methylates H4 K20 and regulates expression of TLR4 target genes such as Cxcl10, IL1a, and Ccl4 [11].

On the other hand, the new stereotypes may refine our understanding of SMYD roles in cancer and cardiovascular disease. Inflammation is at the heart of many chronic diseases including cancer, heart disease, diabetes, and autoimmune disease. Inflammation is a predisposing factor for cancer as inflammation can generate free radicals which can cause DNA damage [15]. It can also contribute to cancer growth by promoting angiogenesis and release proangiogenic cytokines such as TGF-β and IL-6 [16]. However, inflammation in cancer has two faces; which means it can also suppress cancer. Th1-cell-driven inflammation is cytotoxic to cancer cells [17]. Tumor-infiltrated CD8+ lymphocytes are strongly associated with improved survival in patients with triple negative breast cancer (TNBC) [18]. A high CD8/Foxp3 cell ratio also predicted improved prognosis in TNBC patients [18]. This indicates that SMYD3 might be involved in cancer through suppression of cancer immunity and dampen anti-tumor immune response via promoting Foxp3-dependent immune suppressive pathways.

Immunity and inflammation also kindle new hypotheses for how SMYD1 may contribute to cardiovascular disease. SMYD1 was previously linked to end stage heart failure [19]. SMYD1 overexpression in failing hearts is correlated with dysregulation of several important ion channels such as the sodium channel SCN5A and calcium channel CACH2. This correlation suggests SMYD1 contributes to heart failure likely through affecting ion homeostasis of failing hearts. SMYD1 is present in cytotoxic T cells and may be INFγ inducible [14]. Both cytotoxic T cells and INFγ signaling have been linked to heart failure. Myocardial infiltration by donor cytotoxic T-cells led to acute heart failure in a blood stem cell transplanted patient [20]. High levels of INFγ and its responsive genes are strongly associated with congestive heart failure and dilated cardiomyopathy respectively [21]. These together suggest a new possibility that SMYD1 could be involved in heart failure through inflammation and regulating cytotoxic T-cell function and INFγ signaling pathways.

The era of SMYD proteins in immunity has just begun. There are ample opportunities to generate new, intriguing and potentially clinically important discoveries. New research ideas are waiting to be explored. SMYD proteins may be involved in autoimmune disease as they regulate polarization of T-cells and macrophages [11–13]. SMYD proteins may be involved in neurodegenerative disease as persistent neuroinflammation can cause neuronal damage [22]. Of note, all SMYD proteins are expressed in brain; and knockout of SMYD4 and SMYD5 in mice both resulted in significant behavioral phenotypes [23]. SMYD proteins are present in nucleus and cytosol and methylate histone and non-histone proteins. This knowledge signifies us that research efforts should not be limited to the epigenetic mechanisms and non-histone methylation could also play a role. SMYD3 methylates the mitogen-activated protein kinase MAP3K2 [24]. This methylation could link SMYD3 to the proinflammatory NF-κB signaling pathway, as MAP3K2 directly phosphorylates and activates the NF-κB upstream regulator IKK [25]. SMYD2 methylates p53 and retinoblastoma protein (RB) and regulates their transactivation activity [26,27]. Both p53 and RB can regulate IL-6 expression, through which they suppress autoimmunity [28]. This suggests that a combinatorial input from H3K36 methylation and the non-epigenetic pathways determines the final output of the SMYD2-mediated immune response. Therefore, studying non-histone methylation has the potential to reveal new pathways allowing deeper and broader understanding of SMYD proteins in immunity and inflammation.

SMYD proteins have been an exciting field of study due to their involvement in cancer and cardiovascular development. They will continue to be exciting research topics as emerging data are shifting the field from the traditional stereotypes to a new era: immunity and inflammation.
